# Dr. Robert Arthur

**Published:** 1880-07

**Authors:** 


### EDITORIAL, ETC.

*Death of Dr. Robert Arthur.*—The many personal friends, as well as the very great number who knew Dr. Robert Arthur by reputation, were pained to know of his death, which occurred in Baltimore, in June last, of gangrene of the foot, the result of embolism of an artery.

Probably no man in the Dental profession was so widely known by his works as Dr. Arthur, and certainly as a forcible, terse and elegant writer, he had no equal in Dental literature; probably no man was more misunderstood and consequently unappreciated. Modest and diffident even to shyness, he was correspondingly sensitive. Fertile in brain and industrious intellectually, his thoughts always reverted to schemes for the promotionof his chosen and beloved profession. If he formulated theories which would not fairly stand the test of time, or if he originated methods of operation which were not approved by the profession at large, yet those who knew him best—and only those who got close to the man's heart really knew him—felt, even while they might disagree with him, that Robert Arthur's whole soul was in these methods and theories. Not that he wished for position or praise or exaltation above his brethren, but for the elevation of his profession and the benefit of his kind. His heart was in his work most unselfishly and truly. He was probably one of the most unworldly and ideal men of his day,—a day when utilitarianism has its full sway, and when a man's brains are judged by his success financially,—and knew less of the world and its ways, of its practical business ways than almost any of the writer's acquaintance. Large hearted, pure hearted, simple minded, generous, Dr. Arthur found his ideas bandied about as coarsely as jests, his carefully wrought theories belittled by men utterly unable to grasp their extent, and his whole animus treated most ungenerously. If under these circumstances he became perhaps morbidly sensitive, it was not to be wondered at; and if he found that his want of ability to do that which mere handicraft, with mediocre brains, gave to others, and so came of late to isolate himself from his profession, it is not to be wondered at.

People thought him *exclusive*, and sneered. His was simply the *seclusion* of the diffident scholar, pushed on by the great thoughts within, and hindered by the lack of "want of sensibility." And these opposing forces made him what he appeared to many—a failure. An appearance only. For in this world men are judged not so much by what is in them of good or noble or true, but by what they succeed in doing, the gaping crowd not comprehending that the apparent or even real failure of such a man as Robert Arthur is more of a success than many apparently greater successes; and that his noblest success was in keeping strictly before him his one unselfish motive—to leave his profession better than he found it. If, as it is understood, Dr. Arthur died poor, in spite of the eminent opportunity his talents afforded him to become rich, it will not lower him in the eyes of those who knew him best.

The writer can never forget how, as a student of dentistry, his occasional interviews with Dr. Arthur impressed him, and chiefly his earnestness was conspicuous. Whatever else those who failed to accept his peculiar teachings saw in him, they could not help seeing, that he himself thoroughly believed in them, and that not only his great mind was bent upon them but that his whole heart was in them as well. No one could hear him speak and doubt his earnest sincerity. Now that he is dead it will be seen how largely he left the impress of that mind and heart upon his profession. H.

---

We append a few extracts from letters received from contemporaries of Dr. Arthur. The first is from one who, as retiring as Dr. A., begs that his name shall not be used. He says:

I am extremely pained to learn of the death of Dr. Arthur, the eminent professional brother and high-toned gentleman.

It was through his advice that I entered upon my collegiate course, more than a quarter of a century past, in the old Philadelphia College of Dental Surgery, in which institution he was an able and honored professor.

He was always genial and kind, and one of the most modest gentlemen it was ever my good fortune to be acquainted with Though extremely diffident (even to a fault) in the delivery of his attractive lectures, they were so replete in profound thoughts and practical truths, clothed in plain yet choicest expressions, as to appeal at once to the mind and heart of every grateful student before him. As a lecturer he won the admiration of the entire class, and his uniform courtesy and gentleness marked him a general favourite.

When your kind note informed me of his death, I turned at once to his able valedictory address, delivered before our graduating class, Feb'y 28, 1855, and was much impressed with this passage: "My heart swells with gratitude to the Giver of all good, often and often, when I think of the happy circumstances in which a good Providence has placed us, whilst in so many parts of the world, hard, grinding oppression crushes freedom of action, and even freedom of thought." Also with its concluding words: "Always remember this, gentlemen,—it is a great truth, full of consoling power—there is a Providence which regards our eternal good more than our mere temporary comfort, ordering all our circumstances so as best to secure this end. And rest well assured, that if you perform your duties in your profession always under a high sense of what is right, it will lead you to a success higher and more desirable than can be reached in any other way. It will bring you peace of mind in all your relations, and when your career in your profession and in life draws to a close, no accusing ghosts will stand behind you; and you will look, without dismay, towards the more desirable future then opening before you. You will have completed another period of probation, and it will be well if at that commencement, you are prepared, as a just life will best prepare you, to enter upon a career that will never close."

His earthly career has closed—the eternal commencement has come, and the dear old Professor, we may confidently hope, has entered into that rest which remaineth to the people of God.

---

Dr. W. W. H. Thackston writes:

*My Dear Doctor.*—I have rarely been more shocked by any incident or casualty than by this unanticipated death of a very dear friend, Dr. Arthur, for such were the relations which for a quarter of a century and more have existed between Dr. Arthur and myself.

Entering the Profession together when it was in its transitive state from an "occult art" to a liberal department of general science, we necessarily almost drifted together as representatives of a "new departure," animated by a common cause, and encountering common difficulties and obstacles; working in concert and co-operation, a "fellow feeling" drew us together, and that feeling ripened into the warmest personal friendship and esteem—which "grew with our youth," and on my part, and I think on the part of my lamented friend, "strengthened with our declining years." Dr. Mackall, if he be living, and myself, are now the two oldest surviving graduates of a Dental College in the world. I received the third diploma conferred by the "old College," which was the first, and then the only one, of its kind in existence. Drs. Arthur and Mackall preceded me one year; I graduated at the head of my class the following commencement. My fellow graduates have long since passed away, and I begin to feel hoary and in some sense patriarchial. I am painfully admonished that I too am passing away. How much longer will a kind, a merciful and beneficent Providence permit me to "labor in the vineyard?" And how soon may some kind and perhaps too partial friend be lamenting my call to the "great beyond," as we now lament our loved and lost friend, and professional co-laborer?

---

*Lawn Mowers.*—As many of our profession enjoy the pleasures of country life during a portion of the year when business cares can be profitably laid aside, and the system invigorated by the healthful exercise of out door life, we call attention to Hill's Archimedean Lawn Mower as one of the best in the market. Having recently purchased one for ourselves, we have had an opportunity of personally testing the merits of this Lawn Mower and can confidently recommend it for the completeness of its work and its easy working qualities. Different sizes are furnished from 8 to 18 inches for hand machines and from 24 to 32 inches for pony and horse machines. This and another style known as the Charter Oak can be obtained at the manufactory, Colt's Armory, or from Agents everywhere. Address Hill's Archimedean Lawn Mower Co., Hartford, Conn.

